# Quantum walk with coherent multiple translations induces fast quantum gate operations

**DOI:** 10.1038/s41377-025-02106-3

**Published:** 2026-01-01

**Authors:** Yixiang Zhang, Xin Qiao, Luojia Wang, Yanyan He, Zhaohui Dong, Xianfeng Chen, Luqi Yuan

**Affiliations:** 1 State Key Laboratory of Photonics and Communications, School of Physics and Astronomy, Shanghai Jiao Tong University, Shanghai, 200240 China; 2 College of Physics and electronics Engineering, Northwest Normal University, Lanzhou, 730070 China; 3 Collaborative Innovation Center of Light Manipulations and Applications, Shandong Normal University, Jinan, 250358 China; 4 Shanghai Research Center for Quantum Sciences, Shanghai, 201315 China

**Keywords:** Quantum optics, Quantum optics

## Abstract

Quantum walks with one-dimensional translational symmetry are important for quantum algorithms, where the speed-up of the diffusion speed can be reached if long-range couplings are added. Our work studies a scheme of a ring under the strong resonant modulation that can support a discrete-time quantum walk including coherent multiple long-range translations in a natural way along the synthetic frequency dimension. These multiple translation paths are added in a coherent way, which makes the walker evolve under the topological band. Therein, not only the fast diffusion speed is expected, but more importantly, we find that single quantum gate operations can be performed in the quasi-momentum space. In particular, we show the arbitrary single-qubit state preparation and an example of CNOT two-qubit gate with only one time step, dramatically increasing quantum algorithms. Our study uses the modulated ring to provide fast quantum gate operations based on coherent multiple path quantum walk, which may provide unique designs for efficient quantum operations on photonic chips.

## Abstract (web summary)

The strong modulation introduces coherent-multiple-transition into discrete time quantum walks in the frequency space, inducing topological band and fast quantum gate operations.

## Graphical abstract

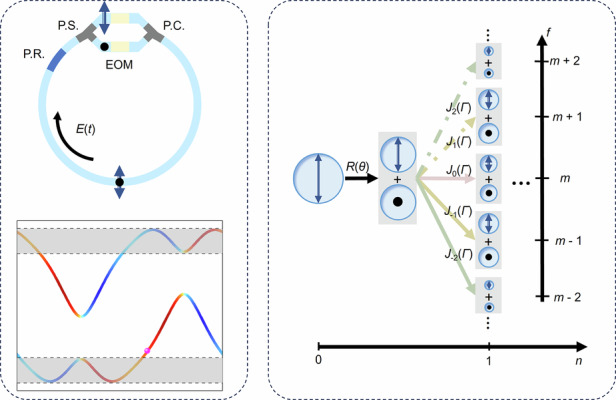

## Introduction

The concept of quantum walk^1–4^ has been developed for various subjects including the quantum algorithm design^3,5–9^, quantum dynamics simulation^10–12^, and topological phases exploration^13–20^. Due to quantum interference, quantum walk displays different behaviors than classical random walk^1,2,21,22^. One of the key features is that the quantum walk spreads quadratically faster than its classical counterparts^21,22^, which makes it possible for realizing a version of the Grover’s search algorithm^23,24^ with a square-root reduction in the execution time compared to classical algorithms^25^. Therefore, it has been well recognized that the quantum speed-up of quantum walk is crucial for quantum computing. Due to the interference nature behind^26^, photonic systems provide important platforms for performing quantum walks, including experiments in bulk optics^27–31^, fiber loops^32–35^, fiber cavities^36^, and integrated photonics^37,38^.

It is worth noting that the efficiency of quantum walk in photonic lattices may be further sped up algorithmically if the long-range coupling between lattice sites is added. Especially for the universal quantum gate designs using quantum walks for qubits on the graph^39–42^, it usually requires complex geometric structures in photonics^43,44^. Hence efficient simplification of a photonic configuration in realizing quantum walk including long-range couplings is essential for realizing quantum computing with desired quantum-gate functionality in integrated photonics.

The synthetic frequency dimension built in rings under dynamic modulations may provide a solution as it can provide artificial lattice model with long-range couplings and may be implemented with recent state-of-art technology on photonic chips^45–49^. Compared with conventional platforms^27–38^, quantum walks in synthetic frequency dimension are less dependent on the spatial scale and have high reconfigurability (see the supplementary note I for details). Although various photonic simulations have been demonstrated in synthetic frequency lattice models^50–52^, current researches mainly focus on the weak modulation limit and hence the models obey the continuous-time Schrödinger equation^51–55^. Moreover, these simulations are mathematically similar with counterparts in other quantum walk models^47–49,56,57^ where nearby hoppings are dominating and long-range couplings are added as an addition.

In this paper, we theoretically study the one-dimensional discrete-time quantum walk (1D DTQW) in the synthetic frequency dimension built by a photonic ring under the strong electro-optic modulation (EOM), where translation operators naturally support coherent multiple long-range transition effects. Such a model can lead to the topological band with not only the enhancement of the walker’s diffusion speed, but also the way for constructing arbitrary single quantum gate operations and preparing single-qubit/two-qubit states in the fast manner. Our work reveals a coherent way of quantum interference with multiple long-range translations in the quantum walk can greatly improve the speed in quantum algorithms.

## Results

### Theoretical model of DTQW

**Fig. 1:**
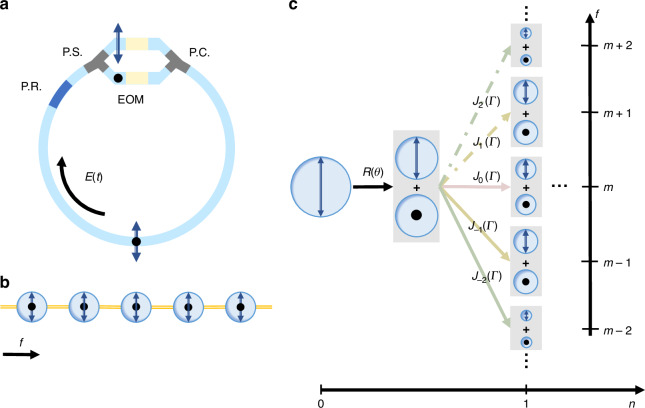
Structure of the ring resonator and the 1D DTQW in the synthetic frequency dimension. **a** Ring resonator with a polarization rotator (P.R.), a polarization splitter (P.S.), two EOMs, and a polarization combiner (P.C.). The dots and black arrows represent *H*- and *V*-polarization respectively. **b** One-dimensional synthetic spin lattice in the frequency dimension. **c** Schematic of 1D DTQW in the synthetic frequency dimension. The area of each circle represents the magnitude of the polarization-dependent amplitude ${\Psi }_{H}(t)$ and ${\Psi }_{V}(t)$

We consider a ring resonator, composed by the photonic waveguide that can support two polarizations of light, as shown in Fig. 1a, where the two orthogonal polarization modes of light (labelled by *H* and *V*) form the pseudo-spin state basis for the electromagnetic waves. A polarization splitter is added into the ring to split light field to *H*- and *V*-polarization components respectively and then each component is propagating through the corresponding polarization-maintaining waveguide branches [see Fig. 1a]. Afterwards, field components from two branches combine into the main section of the ring via a polarization combiner. The similar design has been previously proposed and recently experimentally used to study non-Abelian gauge field^54,55,58^.

The ring supports frequency resonant modes with the free spectral range $\Omega$ for both polarization components if the group velocity dispersion of the waveguide is ignored. Each pair of modes at the same frequency holds an effective spin site. By applying dynamic modulation in each branch, one can connect the spin state at each site in a desired way and hence construct the synthetic spin lattice in the frequency dimension of light (see Fig. 1b). A polarization rotator is added to rotate the polarization of the field inside the ring, i.e., to rotate the spin state in the synthetic lattice.

We here provide the mathematical description of the field dynamics in our designed ring. We first expend the electric field inside the ring as

1 $$\Psi (t)=\mathop{\sum }\limits_{m}{a}_{m}\left(t\right){e}^{{i\omega }_{m}t}$$

Here $\Psi \equiv {[{\Psi }_{H},{\Psi }_{V}]}^{T}$ is the polarization-dependent amplitude of the electric field and ${a}_{m}\equiv {[{a}_{m,H},{a}_{m,V}]}^{T}$ is the polarization-dependent amplitude of the *m*-th resonant mode at the resonant frequency ${\omega }_{m}\equiv {\omega }_{0}+m\Omega$. ${\omega }_{0}$ is a reference resonant frequency in the ring, which can usually be set as zero for simplicity^52^. One can replace the time $t$ by $t=n{T}_{R}+{t}_{f}$, where ${T}_{R}=2\pi /\Omega$, $n$ is a non-negative integer representing the number of round-trips, and ${t}_{f}\in [-{T}_{R}/2,{T}_{R}/2)$ is the travel time of light in each round-trip. The variation of the field after it finishes each round-trip can be described by

2 $$\Psi \left(t+{T}_{R}\right)=\mathop{\sum }\limits_{m}{a}_{m}\left({t+T}_{R}\right){e}^{{i\omega }_{m}t}=D\left(t\right)R\left(\theta \right)\mathop{\sum }\limits_{m}{a}_{m}\left(t\right){e}^{{i\omega }_{m}t}$$

where $R(\theta )$ and $D(t)$ takes the form

3 $$\begin{array}{c}R\left(\theta \right)\equiv {e}^{-i\theta {\sigma }_{y}/2}=\left(\begin{array}{cc}\cos \frac{\theta }{2} & -\sin \frac{\theta }{2}\\ \sin \frac{\theta }{2} & \cos \frac{\theta }{2}\end{array}\right)\end{array}$$

4 $$\begin{array}{c}D\left(t\right)\equiv \left(\begin{array}{cc}{e}^{i\varGamma \cos (\Omega t+{\phi }_{H})} & 0\\ 0 & {e}^{i\varGamma \cos (\Omega t+{\phi }_{V})}\end{array}\right)\end{array}$$

describing the polarization rotation and polarization-dependent modulations in Fig. 1a, respectively. Here $\theta$ is the polarization rotation. Two EOMs are under the resonant phase modulation at the modulation strength $\varGamma$ with different modulation phases ${\phi }_{H}$ and ${\phi }_{V}$. We can extend the polarization-dependent modulations $D(t)$ as

5 $$\begin{array}{l}D\left(t\right)=\left(\begin{array}{cc}{J}_{0}(\varGamma )+\mathop{\sum }\limits_{l=-\infty }^{\infty }{i}^{l}{J}_{l}(\varGamma ){e}^{il(\Omega t+{\phi }_{H})} & 0\\ 0 & {J}_{0}(\varGamma )+\mathop{\sum }\limits_{l=-\infty }^{\infty }{i}^{l}{J}_{l}(\varGamma ){e}^{il(\Omega t+{\phi }_{V})}\end{array}\right)\end{array}$$

${J}_{l}(\varGamma )$ is the $l$-th order Bessel function. By taking Eq. (3) and Eq. (5) into Eq. (2), we have

6 $$\begin{array}{l}{a}_{m}\left(t+{T}_{R}\right)={J}_{0}\left(\varGamma \right)R\left(\theta \right){a}_{m}\left(t\right)+\mathop{\sum }\limits_{l=-\infty }^{\infty }{i}^{l}{J}_{l}\left(\varGamma \right)\left[{d}_{l}R\left(\theta \right){a}_{m-l}\left(t\right)+{d}_{-l}R\left(\theta \right){a}_{m+l}\left(t\right)\right]\end{array}$$

7 $${d}_{l}=\left(\begin{array}{cc}{e}^{il{\phi }_{H}} & 0\\ 0 & {e}^{il{\phi }_{V}}\end{array}\right)$$

This system can be viewed as a quantum walk process described by the unitary step operator. In the constructed synthetic frequency lattice, the basis vector is ${|m},p\rangle$, where $m$ is the coordinate of the walker in the synthetic frequency dimension, and $p$ is the spin state (the coin state^3^) based on ${|H}\rangle$ and ${|V}\rangle$. Therefore, by placing Eq. (1) into Eq. (2) and keeping terms at the same ${\omega }_{m}$, the unitary step operator $U$ after each roundtrip reads $U={TR}(\theta )$, where $T$ denotes a polarization-dependent translation operator in synthetic space from Eq. (6):

8 $$T=\mathop{\sum }\limits_{m,l=-\infty }^{\infty }{i}^{l}{J}_{l}({\Gamma }){e}^{il{\phi }_{H}}|m+l\rangle \langle m|\otimes |H\rangle \langle H|+\mathop{\sum }\limits_{m,l=-\infty }^{\infty }{i}^{l}{J}_{l}({\Gamma }){e}^{il{\phi }_{V}}|m+l\rangle \langle m|\otimes |V\rangle \langle V|$$

The operator $U$ reflects the physics of the original periodically driven system in Eq. (2). The dynamics of such quantum walk process can be understood by the schematic in Fig. 1c, where the field at the $m$-th resonant mode gets the rotation operation ($R$) and then undergoes the polarization-dependent translation operation ($T$) for a single roundtrip. The walker (the wave function of the field in the synthetic frequency lattice) transit to adjacent modes under the weak modulation strength $\varGamma$ may derive to the continuous-time Schrödinger equation in previous works^51–53^. Nevertheless, once $\varGamma$ is large enough, the model goes beyond the weak modulation limit and becomes 1D DTQW (see the supplementary note II for details). Under this strong modulation limit, long-range transitions spaced by $l$ sites with the coupling strength ${J}_{l}(\varGamma )$ are naturally included, as shown in Fig. 1c.

To understand the proposed model supports the quantum walk with multiple transitions in a coherent way, we transform $T$ into quasi-momentum space which is reciprocal to the frequency and in the unit of time, and obtain

9 $${U}_{k}={\int }_{\mathrm{BZ}}dk\left(\begin{array}{cc}{e}^{i\varGamma \,\cos (k+{\phi }_{H})}\cos \,\frac{\theta }{2} & -{e}^{i\varGamma \,\cos (k+{\phi }_{H})}\sin \,\frac{\theta }{2}\\ {e}^{i\varGamma \,\cos (k+{\phi }_{V})}\sin \,\frac{\theta }{2} & {e}^{i\varGamma \,\cos (k+{\phi }_{V})}\cos \,\frac{\theta }{2}\end{array}\right)\otimes |k\rangle \langle k|$$

where $k$ is the quasimomentum and is exactly ${t}_{f}$^59^. By applying the Floquet band theory^60^ and using $U={e}^{-i{H}_{{\rm{eff}}}{T}_{R}/\hslash }$, we obtain an effective Hamiltonian in quasimomentum space:

10 $${H}_{{\rm{eff}}}=\frac{\varOmega }{2\pi }{\int }_{{\rm{BZ}}}dk[E(k){\bf{n}}(k)\cdot {\boldsymbol{\sigma }}]\otimes |k\rangle \langle k|$$

Here $E(k)$ is the quasienergy satisfies

11 $$\begin{array}{c}\cos E\left(k\right)=\pm \cos \left[\frac{\varGamma }{2}\left(\alpha +\beta \right)\right]\cos \frac{\theta }{2}\cos \left[\frac{\varGamma }{2}\left(\alpha -\beta \right)\right]\mp \sin \left[\frac{\varGamma }{2}\left(\alpha +\beta \right)\right]\sin \left(k,{\phi }_{H},{\phi }_{V}\right)\end{array}$$

where $$\alpha =\cos \left(k+{\phi }_{H}\right)$$, $$\beta =\cos \left(k+{\phi }_{V}\right)$$ and

12 $$\begin{array}{c}\sin \left(k,{\phi }_{H},{\phi }_{V}\right)=\sqrt{1-{\left\{\cos \left[\frac{\varGamma }{2}(\alpha -\beta )\right]\cos \frac{\theta }{2}\right\}}^{2}}\end{array}$$

${\boldsymbol{\sigma }}=({\sigma }_{x},{\sigma }_{y},{\sigma }_{z})$ and ${\bf{n}}=({n}_{x},{n}_{y},{n}_{z})$ are the Pauli matrix vector and polarization eigenstate vector, respectively (see the supplementary note III for details). $\hslash =1$ for the simplicity.

**Fig. 2:**
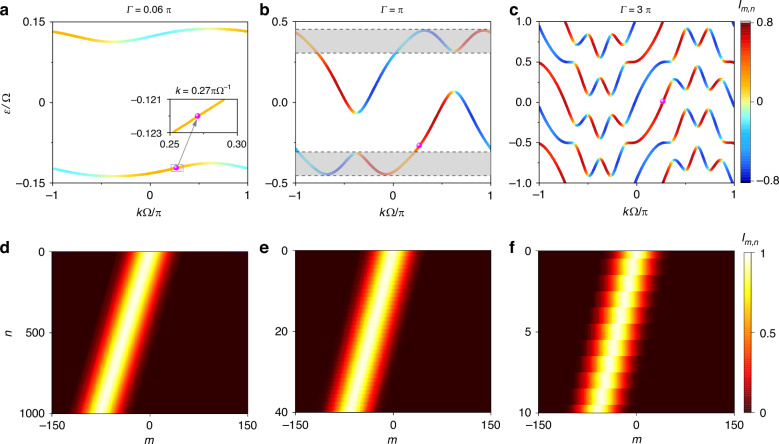
Quasienergy spectrums and evolutions of eigenstates. Quasienergy spectrum of the Hamiltonian *H*_eff_ with $(\theta ,{\phi }_{H},{\phi }_{V})=(-\pi /\mathrm{2,0,3}\pi /4)$ and different modulation intensities: **a** $\varGamma =0.06\pi$, **b** $\varGamma =\pi$**, c** $\varGamma =3\pi$. The colorbar specifies the projection of eigenstates on *H*- (or *V*-) polarization with the value up to $1(-1)$. **d**–**f** Evolutions of eigenstates excited at quasi-momentum $k=0.27\pi {\Omega }^{-1}$ in simulations with different $\varGamma$ in simulations

We plot the quasienergy spectra from ${H}_{{\rm{eff}}}$ with various choices of $\varGamma$ in Fig. 2a–c. For Fig. 2a, we choose $\varGamma =0.06\pi$, i.e., the weak modulation, with parameters $\theta =-\pi /2$, ${\phi }_{H}=0$, and ${\phi }_{V}=3\pi /4$. One can see the typical topological edge modes associated to the quantum Hall ladder^61^, where the spectrum exhibits one-way dispersion for the projection onto one polarization (see the supplementary note IV for details). On the upper band, the *H*- (*V*-) polarization is locked with the positive (negative) dispersion, and vice versa for the lower band, showing the polarization and momentum locking. Note the range of each band in the spectrum is $0.02\Omega$ due to the small $\varGamma$. Such range is greatly enlarged once $\varGamma$ becomes larger [see Fig. 2b and c]. In Fig. 2b with $\varGamma =\pi$ in the strong modulation limit, the model with coherent multiple transitions supports bands, expanding over the spectral first Brillouin zone (FBZ) $\varepsilon \in [-\mathrm{0.5,0.5}]\Omega$, with more concentration occupied on each polarization. Moreover, at a portion of $k$, the one-way edge modes on one band projected on the same polarization are paired with opposite dispersions for a given $\varepsilon$ [see the shadow region in Fig. 2b], which is fundamentally different from that in Fig. 2a, as in this shadow region, the excited edge mode may be scattered to the one at the opposite direction in principle. However, one can see that there is still part of edge modes that are free of such pairing. In addition, large $\varGamma$ results in greater steepness, which leads to the larger speed of the quantum walker evolution. Lastly, if $\varGamma$ is further enlarged, the bands outside of the spectral FBZ get into FBZ, for example as shown in Fig. 2c with $\varGamma =3\pi$. The spread speed of the quantum walker gets further enhanced and the shadow region disappears as no pairing of modes at the same polarization but opposite dispersions on one band. Here we give several additional notes. Firstly, the pairing is limited in edge modes on the same band. In principle, the two separate bands are protected by different topological invariants if bulks are introduced, for example, by using multiple ($> 2$) ring resonators in the spatial space (see the supplementary note V for details). Secondly, for one band in Fig. 2c, the edge mode projected onto *H*- (or *V*-) polarization supports three portions with negative dispersion and two portions with positive dispersion, and vice versa. Therefore, the pairing here can be multiple modes with opposite dispersions.

In simulations, we excite the walker using a Gaussian-shape wave packet ${\bf{s}}(m)={e}^{-{m}^{2}/{\Delta }^{2}}{e}^{-{ikm}\Omega }{{\bf{e}}}_{{\bf{s}}}$ in the synthetic lattice, where ${{\bf{e}}}_{{\bf{s}}}={\left(\langle {H|}{\bf{n}}(k)\cdot {\boldsymbol{\sigma }}\rangle ,\langle {V|}{\bf{n}}(k)\cdot {\boldsymbol{\sigma }}\rangle \right)}^{T}$ carries the initial polarization information. We apply the unitary step operator $U$ to the initial state ${|s}\rangle$ to simulate light circulating in the ring for one roundtrip. We choose corresponding ${{\bf{e}}}_{{\bf{s}}}$ and $\Delta =25$, $k=0.27\pi {\Omega }^{-1}$ to excite the eigenstates labelled by pink dots in Fig. 2a–c and plot the wave function distribution $P(m,n)={\sum }_{p}{|\langle m,{p|}{U}^{n}|{\bf{s}}\rangle |}^{2}$ after $n$-th step (roundtrip) in Fig. 2d–f. One sees all evolutions exhibit unidirectional frequency conversion with similar patterns, but the one with larger $\varGamma$ shows significant faster evolution speed of the quantum walker in the synthetic frequency lattice.

**Fig. 3:**
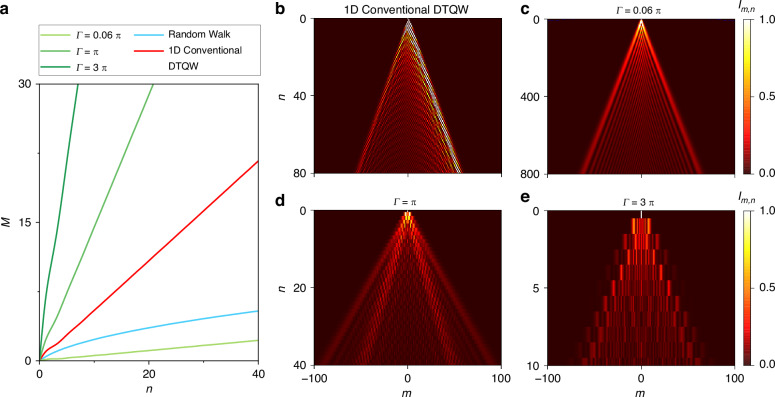
Diffusion distances of classical random walk and DTQW. **a** Diffusion distances calculated in the classical random walk (blue), 1D conventional DTQW (red), and 1D DTQW in the frequency dimension with three different $\varGamma$ (light green, green, and dark green, respectively) versus step (roundtrip) number $n$. Evolutions of **b** 1D conventional DTQW, and **c**–**e** 1D DTQW in the synthetic frequency dimension with **c** $\varGamma =0.06\pi$, **d** $\varGamma =\pi$, **e** $\varGamma =3\pi$ in simulations

To further compare the speed of the wave-function diffusion between our model, 1D conventional DTQW^3^, and classical random walk^62^, we study the diffusion of the wave function with the initial single-site excitation, i.e., with the initial state $|{\varphi }_{0}\rangle =|0\rangle \otimes {|H}\rangle$. The diffusion distance $M(n)\equiv \sqrt{{\sum }_{m,p}{m}^{2}{|\langle m,{p|}{U}^{n}|{\varphi }_{0}\rangle |}^{2}}$ is defined, which represents the most probable position of the quantum walker after $n$ steps. Figure 3 shows the comparison with simulation results of diffusion dynamics of the quantum walker. One sees that the conventional DTQW spreads quadratically faster than the classical random walk in one dimension. Interestingly, the diffusion in the synthetic frequency lattice depends largely on $\varGamma$. For $\varGamma =0.06\pi$, the model is in the weak modulation limit and exhibits the tight-binding feature with only the nearest-neighbor connectivity^51,52^. The diffusion speed of the walker is slower than that of classical random walks. However, as $\varGamma$ grows to $\pi$ and $3\pi$, the slopes of quasienergy spectrums become sharper and simulation results show much faster diffusion speeds of the walker exceeding that of DTQW. According to the fitting of simulation results, we find that $M\propto n$ for DTQWs in frequency dimension, and the slope $M/n$ changes with the modulation strength $\varGamma$, satisfying $M/n=1.38/\pi \varGamma$. As comparisons, for 1D random walk and 1D conventional DTQW, there are $M=\sqrt{n}$ and $M=0.54n$, respectively. One can see that our model in the strong modulation case has a significant speed advantage (see the supplementary note VI for details). Especially, when $\varGamma =3\pi$, the diffusion speed is accelerated to about 8 times of the speed in DTQW. Moreover, we also find that the wave function distribution during evolution in synthetic lattice is symmetric, as the translation operator $T$ in our model translates two polarization modes in both directions with the same coupling strength. Besides, by adding perturbations into simulations, we verify the robust quantum walks in our model against small disorders (see the supplementary note VII for details).

The acceleration of the diffusion speed with large $\varGamma$ in our model thanks to the simultaneous translation of the walker over multiple frequency sites, i.e., the diffusion over long distances, but in a coherent way. Moreover, the quantum walk transport in our proposal still holds high efficiency, proven by the calculation of the return probability^63,64^ (see the supplementary note VIII for details). With the help of such acceleration, the conversion of the quantum state wave function in the frequency dimension becomes faster, which may provide the significant speed-up of various quantum algorithms based on quantum walks.

### Quantum gate operations

From the coherent multiple long-range translations [Eq. (8)], the evolution operator in Eq. (9) and its corresponding band in Eq. (11) provide a way for implementing quantum gate operations in one time step under specific parameters. We consider basic quantum operations, such as *X*, *Y*, *Z* gates and Hadamard (*H*) gate, phase-shift (${R}_{z}$) gate^65^. The qubit can be assigned at a given $k$ in quasi-momentum space $|{\phi }_{1},{\phi }_{2}\rangle \otimes {|k}\rangle$, where $|{\phi }_{1},{\phi }_{2}\rangle =\cos ({\phi }_{1}/2){|H}\rangle +\sin ({\phi }_{1}/2){e}^{i{\phi }_{2}}{|V}\rangle$, which corresponds to the wave function distribution of the field in the lattice along the frequency dimension after the Fourier transform. Specific modulations are then taken to convert the wave function distribution into the target qubit’s distribution on the lattice, which finally leads to a final state in quasi-momentum space. Such entire process can give a quantum operation in one time step (i.e., the field circulates for one roundtrip).

We take the building of the *X* gate as an example by setting $\theta =\pi$, $\varGamma \cos (k+{\phi }_{H})=\pi$ and $\varGamma \cos (k+{\phi }_{V})=0$, so the corresponding operator $U_k\equiv \int_{BZ} {dk}{M}_{U}\otimes {|k}\rangle \langle {k|}$ has $${M}_{U}=\left(\begin{array}{cc}0 & 1\\ 1 & 0\end{array}\right)$$ for qubits $|{\phi }_{1},{\phi }_{2}\rangle \otimes {|k}\rangle$. In reality, one can realize the condition $\theta =\pi$ by setting the rotational angle in an on-chip polarization rotator^66^ to $\pi$. The quasi-momentum $k$ is calibrated by the moment the photon is sent to the ring. Then to meet the conditions $\varGamma \cos (k+{\phi }_{H})=\pi$ and $\varGamma \cos (k+{\phi }_{V})=0$, we set the modulation strength $\varGamma =\pi$, and find the modulation phase ${\phi }_{H}$ and ${\phi }_{V}$ as $-k$ and $(\pi /2-k)$. By doing so, we obtain the necessary modulation signals $\varGamma \cos (k+{\phi }_{H})$ and $\varGamma \cos (k+{\phi }_{V})$ in two EOMs. The simulation is then taken in the synthetic lattice by taking the initial excitation with $\Delta =200$, $k=2\pi /3\Omega$, and ${{\bf{e}}}_{{\bf{s}}}={(\mathrm{1,0})}^{T}$ or ${(\mathrm{0,1})}^{T}$. After one roundtrip, we convert the resulting field distribution to the $k$ space, which gives the output qubit state. The operation matrix can then be bulit from input and output qubit states. Finally, the fidelity of this quantum gate is calculated by ${F}_{g}=1-{{||}{M}_{t}-{M}_{o}{||}}_{{\rm{HS}}}^{2}=1-{\rm{Tr}}\left[\left({M}_{t}^{\dagger }-{M}_{o}^{\dagger }\right)\left({M}_{t}-{M}_{o}\right)\right]$ between the operation matrix ${M}_{o}$ and the target matrix ${M}_{t}$, which results in ${F}_{g}=1$. Here $||{M}_{t}-{M}_{o}|{|}_{{\rm{HS}}}^{2}$ is the Hilbert-Schmidt norm of ${M}_{t}-{M}_{o}$. Similarly, other gates can be obtained by varying parameters [see Table 1].

**Table 1:** Parameters used for realizing quantum gates in quasi-momentum space

	$\theta$	$\varGamma \cos (k+{\phi }_{H})$	$\varGamma \cos (k+{\phi }_{V})$	${M}_{U}$
*X* gate	$\pi$	$\pi$	0	$$\left(\begin{array}{cc}0 & 1\\ 1 & 0\end{array}\right)$$
*Y* gate	$\pi$	$\frac{\pi }{2}$	$\frac{\pi }{2}$	$$\left(\begin{array}{cc}0 & -i\\ i & 0\end{array}\right)$$
*Z* gate	0	0	$\pi$	$$\left(\begin{array}{cc}1 & 0\\ 0 & -1\end{array}\right)$$
*H* gate	$-\frac{\pi }{2}$	0	$\pi$	$$\left(\begin{array}{cc}1 & 1\\ 1 & -1\end{array}\right)$$
${R}_{z}$ gate	0	0	$\varphi$	$$\left(\begin{array}{cc}1 & 0\\ 0 & {e}^{i\varphi }\end{array}\right)$$

The coherent nature of DTQW with multiple long-range translations leads to the faster diffusion in quantum walk, which makes the wave function of the qubit (walker) spread over a larger distance in one step (roundtrip) of the DTQW. Therefore, the wave function of the qubit can approach the target qubit’s distribution in the frequency dimension within much fewer steps, indicating the speed-up of the quantum gate preparation and reduce its time steps into $1$ step. As the comparison, for the same model but under the weak modulation, e.g., $\varGamma =0.01\pi$, a phase-shift gate operation may be achieved by using Eq. (9) with the choice of $\cos (k+{\phi }_{H})=0$, $\cos (k+{\phi }_{V})=-1$, and $\theta =0$. However, as the spread distance in one step is small here, it needs 25 steps to implement the phase-shift gate operation (see the supplementary note IX for details).

Arbitrary single qubit state can then be prepared using the proposed quantum gates. We use two *H* gates and two ${R}_{z}$ gates to illustrate the representative process:

13 $$\begin{array}{l}|{\phi }_{1},{\phi }_{2}\rangle \otimes {|k}\rangle ={U}_{{R}_{z}}\left({\phi }_{2}+\frac{\pi }{2}\right){U}_{H}\end{array}{U}_{{R}_{z}}({\phi }_{1}){U}_{H}{|H}\rangle \otimes {|k}\rangle$$

**Fig. 4:**
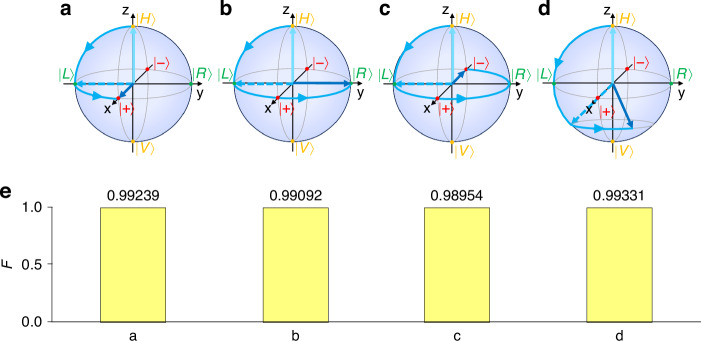
Illustrations of single qubit state preparations using multiple single quantum gates. The targeting single qubit states are **a** $|0.5\pi ,0\rangle$, **b** $|0.5\pi ,0.5\pi \rangle$, **c** $|0.5\pi ,\pi \rangle$ and **d** $|0.75\pi ,0.25\pi \rangle$ respectively. **e** The fidelity between these four target single qubit states and their respective output qubit states

These four gates can change the state ${|H}\rangle$ to arbitrary qubit state $|{\phi }_{1},{\phi }_{2}\rangle$. The fidelity of quantum states is calculated by $F={\left|\left\langle {\psi }_{o}|{\psi }_{t}\right\rangle \right|}^{2}$ between the output qubit and the target qubit, where $|{\psi }_{o}\rangle$ and $|{\psi }_{t}\rangle$ are wavefunctions of these two qubits in the frequency space. Four examples are performed with multiple single quantum gate operations illustrated in Fig. 4, where ${U}_{H}{U}_{{R}_{z}}({\phi }_{1}){U}_{H}$ rotates the initial state ${|H}\rangle$ by ${\phi }_{1}$ about the $x$ axis, and then ${U}_{{R}_{z}}({\phi }_{2}+\frac{\pi }{2})$ rotates it by ${\phi }_{2}$ about the $z$ axis to achieve the desired state $|{\phi }_{1},{\phi }_{2}\rangle$. As results, high fidelity in simulations is shown in Fig. 4e. One can see that the fidelities decrease in Fig. 4a–c, which is caused by the finite frequency range we used in simulation and the strong modulation (see the supplementary note X for details). Besides, considering the losses form the coupling between the resonant ring and the waveguide, the polarization rotator, the polarization splitter, and the EOMs in potential experiments, the fidelity is estimated to $F\sim 97 \%$ (see the supplementary note XI for details).

**Fig. 5:**
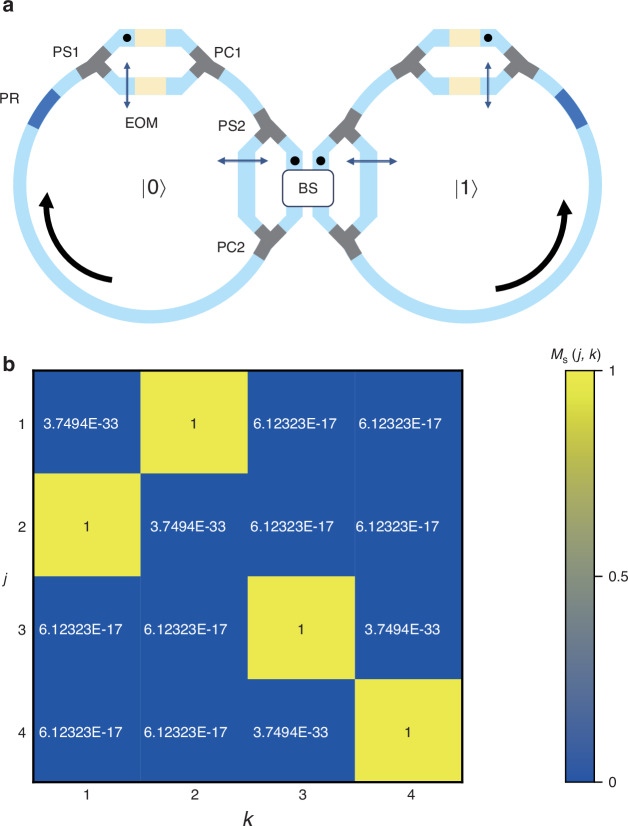
The CNOT gate and reconstructed matrix ${M}_{s}$. **a** A CNOT gate consisting of two ring resonators with polarization rotator (PR), polarization splitter (PS), EOMs, polarization combiner (PC), and a 1:1 beam splitter (BS). The dots and black arrows represent *H*- and *V*- polarization respectively. **b** The reconstructed matrix from simulation results

Our model may be further extended to implement multi-qubit gates by introducing other degrees of freedom^42^. As an example, we extend our proposal including the position degree of freedom in real space to construct a CNOT gate. We implement the CNOT gate by adding another identical ring resonator, as shown in Fig. 5a. We take the left and right rings as qubit $|0\rangle$ and $|1\rangle$, respectively. The quantum state for the walker with *H*- (*V*-) polarization in the left ring is $({|H}\rangle \otimes {|k}\rangle )\otimes |0\rangle$ [$({|V}\rangle \otimes {|k}\rangle )\otimes |0\rangle$], and for the walker in the right is $({|H}\rangle \otimes {|k}\rangle )\otimes |1\rangle$ [$({|V}\rangle \otimes {|k}\rangle )\otimes |1\rangle$]. When the light passes through PS2, the light in polarization state ${|V}\rangle$ is coupled to the other ring through the beam splitter, while the light in ${|H}\rangle$ stays in the original ring. This model supports the two-qubit quantum state in the form:

14 $${M}_{{\rm{CNOT}}}=\left(\begin{array}{cccc}1 & 0 & 0 & 0\\ 0 & 1 & 0 & 0\\ 0 & 0 & 0 & 1\\ 0 & 0 & 1 & 0\end{array}\right)$$

which is consistent with the matrix form of the CNOT gate. In this way, we extend our model beyond the single-qubit gate. We can in-principle increase the number of resonant rings and arrange them in the way similar to literatures^42,67,68^, thus extending our system to the multi-qubit case. Considering the fidelity reduction when inducing new resonator into the system, in practical applications, a maximum of 5 resonant rings can be introduced (see the supplementary note XII for details).

We then apply an *X* gate, a CNOT gate, and an *X* gate operation in a sequence on any qubit, whose operation matrix is:

15 $$\begin{array}{c}{M}_{s}={M}_{X}{M}_{{\rm{CNOT}}}{M}_{X}=\left(\begin{array}{cccc}0 & 1 & 0 & 0\\ 1 & 0 & 0 & 0\\ 0 & 0 & 1 & 0\\ 0 & 0 & 0 & 1\end{array}\right)\end{array}$$

In the simulation, we input four linearly independent 4D vector and calculate the output vectors. Based on these four sets of inputs and outputs, we can reconstruct the operation matrix ${M}_{s}$, as shown in Fig. 5b, which is closely matched with the Eq. (15). It shows that our model still has high accuracy in the multi-qubit case.

## Discussion

In summary, we propose a method to implement a 1D DTQW system in a synthetic frequency lattice. With strong modulations, we break the weak coupling limit and show multiple long-range couplings can transport the quantum walker largely separated apart, following the topological band in a coherent way and with the faster diffusion speed. Compared with ref. ^49^, we use only one EOM for inducing long-range couplings to preserve the specific feature if the dynamic in the strong modulation case, and also potentially simplify the experiment (see the supplementary note XIII for details). The theoretical analysis method of discrete-time quantum walks used in this proposal also fills in the absence of analysis methods for modelling synthetic frequency dimension in the strong modulation limit. Moreover, this model provides the way for constructing single quantum gates in the quasi-momentum space and then further shows the capability for preparing arbitrary single-qubit state as well as multi-qubit gates.

Our proposal is based on a geometrically simple but experimental feasible ring resonator design^55,61,69–71^, with generalization towards integrated photonic chips^45,47–49,72–74^, whose requirements for initial state preparations and measurements are experimentally achievable from state-of-art technologies^48^ for both the single-qubit gates and multi-qubit gates operation (see the supplementary note XIV for details). For example, such a ring resonator can be fabricated on the thin-film lithium niobite chip as a microresonator^48^ (see the supplementary note XV for details). Besides, our proposal can be extended to a two-dimension DTQW by adding another pair of branches with EOMs to fold the frequency dimension^75–77^ (see the supplementary note XVI for details). It can also be extended to two photons case with photon-photon interaction by inducing ${\chi }^{(3)}$ nonlinear process (see the supplementary note XVII for details). Both of examples show the strong scalability of our proposal. Our model with the band under large $\varGamma$ makes quantum gate operations to be implemented with 1 step (roundtrip), thus minimizing the impact on the performance from loss. The strong-modulation induced quantum walk with intrinsic coherent long-range couplings does not require additional modulation signals at higher frequencies, which potentially provides the important photonic on-chip applications with the quantum algorithm speed-up and the quantum circuit simplification.

## Materials and methods

Supplementary Information is available for this paper at 10.1038/s41377-025-02106-3 for details of the theoretical model, numerical simulation, initial state preparations and measurements, ring resonator, 2D DTQW, and photon-photon interaction.

## Supplementary information

Supplmentary Material for: Quantum walk with coherent multiple translations induces fast quantum gate operations

## Data Availability

The data presented in the manuscript is available from the corresponding author upon reasonable request.
