# Supplementary material for: Quantum walk with coherent multiple translations induces fast quantum gate operations
Source: Light Sci Appl. 2026 Jan 1;15:1. doi: 10.1038/s41377-025-02106-3 (PMC12756317; doi:10.1038/s41377-025-02106-3)
Supplement: Supplementary file 1 — Supplmentary Material for: Quantum walk with coherent multiple translations induces fast quantum gate operations [file 41377_2025_2106_MOESM1_ESM.pdf]

Supplmentary Material for: Quantum walk with coherent multiple translations induces fast quantum gate operations

Yixiang Zhang[1,†], Xin Qiao[1,2,†,*], Luojia Wang[1], Yanyan He[1], Zhaohui Dong[1], Xianfeng Chen[1,3,4], Luqi Yuan[1,*]

[1]*State Key Laboratory of Photonics and Communications, School of Physics and Astronomy, Shanghai Jiao Tong University, Shanghai 200240, China*

[2]*College of Physics and Electronics Engineering, Northwest Normal University, Lanzhou 730070, China*

[3]*Collaborative Innovation Center of Light Manipulations and Applications, Shandong Normal University, Jinan 250358, China*

[4]*Shanghai Research Center for Quantum Sciences, Shanghai 201315, China*

[†]*These authors contributed equally.*

[*]*Corresponding authors: Xin Qiao (qiaox@nwnu.edu.cn), Luqi Yuan (yuanluqi@sjtu.edu.cn)*

I. THE COMPARISON BETWEEN QUANTUM WALKS IN SYNTHETIC FREQUENCY LATTICES AND IN CONVENTIONAL PLATFORMS

In bulk optics, one can use a calcite beam-displacer [1] or liquid-crystal (LC) device [2–4] combined with a wave plate to implement one step of conventional discrete time quantum walks (DTQWs). For quantum walks in fiber loops [5–8], a fiber loop with two different-length branches and a polarization splitter can achieve conventional DTQWs in a time multiplexed network. The case in fiber cavities [9] is similar to fiber loops, except that the two branches are replaced by two coupled asymmetric fiber ring resonators. In integrated photonics, a normal method for constructing conventional DTQWs is to utilize integrated waveguides [10, 11]. Since introducing long-range transitions is challenging on conventional platforms, all the translation operations implemented in these platforms mostly consider the hopping to the nearest neighboring positions.

As the comparison, due to the use of dynamic modulation of synthetic frequency dimensions to implement quantum walks (QWs), our system offers richer reconfigurability under external modulation. For example, in the weak modulation limit, one can apply signal including sinusoid functions at multiple frequencies on EOM to introduce long-range coupling into the frequency lattice, enabling the walker to achieve long-range hopping. Meanwhile, in the strong modulation case, the coherent multiple long-range couplings appear in the lattice naturally, not only increasing the diffusion speed of QWs, but also emerging new physical phenomena.

Besides, compared with bulk optics, fiber loops, and fiber cavities, QWs in synthetic frequency lattices combined with integrated photonics can be constructed with single or limited number of resonant rings, therefore being less dependent on the spatial scale and relatively suitable for the application in the photonics chip regime, where the spatial footprint is limited. In comparison to integrated waveguides, QWs in synthetic frequency lattices can offer programmable devices for integrated quantum computing, because of the high tunability of synthetic frequency dimension.

II. THE DIFFERENT DYNAMICS THEORIES OF THE SYNTHETIC FREQUENCY LATTICE UNDER WEAK AND STRONG MODULATION

The theories describing the dynamics of the synthetic frequency lattice under weak and strong modulation are completely different. The previous papers [13, 14, 17] mainly focus on the weak modulation limit and hence the model obeys continuous-time Schrödinger equation. In other words, under the weak modulation limit, one assumes that the variation of each frequency mode of the field after it passes EOM is small so such variation of the field circulates inside the ring for a single roundtrip can be re-written as the first-derivative over the time:

$$a_m(t+T_R) - a_m(t) \equiv T_R \frac{\partial a_m(T)}{\partial T}, \tag{S1}$$

leading to the wave equation describing the evolution of the field over continuous time variable, i.e., the continuous-time Schrödinger equation.

On the other hand, once we extend the model to the strong modulation regime, the derivation on previous literatures to the continuous-time Schrödinger equation is no longer valid, as the variation of the field after it passes EOM is not small. Here, because each frequency mode of the field changes largely after it passes EOM, the variation of the field is then discrete for each time it circulating one roundtrip inside the ring. Therefore, one has to treat the model in a discrete way. Here we also take our model as an example and present the detailed derivations to comparatively reflect the difference.

Beginning with the Eq. (6) - (7) in our manuscript, under the weak modulation limit where $\mathit{\Gamma} \ll 1$ and $\theta \to 0$, the amplitude of a_m changes very slowly over time, so we can write $\partial_t a_m \equiv [a_m(t+T_R) - a_m(t)]/T_R$. After ignoring the higher-order Bessel terms, we get

$$i\partial_t a_m = -\frac{J_1(\mathit{\Gamma})}{T_R}[d_1 R(\theta) a_{m-1} + d_{-1} R(\theta) a_{m+1}], \tag{S2}$$

which is the continuous-time Schrödinger equation describing the dynamics of our model under weak modulation.

As the comparison, in the strong modulation case, the approximation $\partial_t a_m \equiv [a_m(t+T_R) - a_m(t)]/T_R$ does not hold, since $[a_m(t+T_R) - a_m(t)]$ is no longer a small quantity. Therefore, the previous methods cannot describe the system and we need a new approach, i.e., the DTQW theory shown in our manuscript fills in the absence of analysis methods for modelling synthetic frequency dimension in the strong modulation limit, which is one of the

innovations of our manuscript compared to previous articles.

III. EIGENSTATE VECTOR OF 1D DTQW IN THE FREQUENCY DIMENSION

Here we explain the specific form of $\mathbf{n} = (n_x, n_y, n_z)$ in the main text. $\mathbf{n} = (n_x, n_y, n_z)$ is the polarization eigenstate vector of 1D frequency-dimension DTQW system on the Bloch sphere, and it takes the form

$$\begin{aligned} n_x =& \frac{\sin\frac{\theta}{2}\sin\left[\frac{\Gamma}{2}(\alpha-\beta)\right]}{\sin(k,\phi_H,\phi_V)}, \\ n_y =& \frac{\sin\frac{\theta}{2}\cos\left[\frac{\Gamma}{2}(\alpha-\beta)\right]}{\sin(k,\phi_H,\phi_V)}, \\ n_z =& \frac{-\cos\frac{\theta}{2}\sin\left[\frac{\Gamma}{2}(\alpha-\beta)\right]}{\sin(k,\phi_H,\phi_V)}, \end{aligned} \tag{S3}$$

where $\alpha = \cos(k+\phi_H)$, $\beta = \cos(k+\phi_V)$ and

$$\sin(k,\phi_H,\phi_V) = \sqrt{1-\left\{\cos\left[\frac{\Gamma}{2}(\alpha-\beta)\right]\cos\frac{\theta}{2}\right\}^2}. \tag{S4}$$

So Eq. (S3) is the specific form of $\mathbf{n} = (n_x, n_y, n_z)$ in the main text.

IV. THE TOPOLOGICAL EDGE MODES OF HALL LADDER IN SYNTHETIC FREQUENCY DIMENSION

To clarify the topology property in our system, we provide further discussion here. As shown in Fig. S1a, in the weak modulation limit, our proposal is equivalent to a Hall ladder in the synthetic frequency dimension [15] with effective magnetic field flux $\phi_e = \phi_H - \phi_V$, which has been formally shown to reproduce the eigenstates of the topological chiral edge modes of a 2D quantum Hall insulator described by the Hofstadter model [16]. Since the entire bulk lattice sites are removed, there exists no bulk band and also the topological invariant cannot be calculated, while the topological edge modes are retained. When under the strong modulation, although the equivalent Hall ladder model is no longer valid due to long-range connectivities, these edges modes are partially preserved [see Fig. 2b and Fig. 2c in the manuscript].

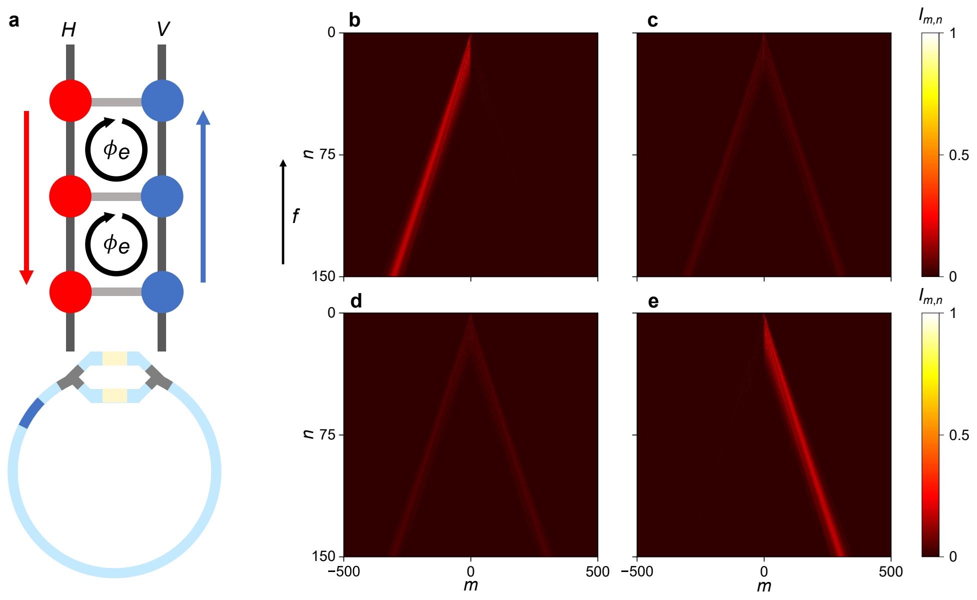

FIG. S1. **The Hall ladder in the synthetic frequency dimension and the evolutions of edge modes. a,** The equivalent Hall ladder and the excitation of the topological chiral edge modes of a 2D quantum Hall insulator in the weak modulation limit. The red (blue) circles represent the frequency modes with H- (V-) polarization. The red and blue arrows represent the direction of topological chiral currents. **b-e,** Evolutions of the modes with different polarizations, being excited by different excitation source in simulations: **b,** H-polarized edge modes, excited by H-polarized pulse, **c,** V- polarized modes, excited by H-polarized pulse, **d,** H-polarized modes, excited by V-polarized pulse, and **e,** V-polarized edge modes, excited by V-polarized pulse.

We verify the edge mode distribution with the pulse excitation and strong modulation strength $\Gamma = \pi$ in simulations. Here, we excite the walker using Gaussian-shape pulse $\mathbf{s}(n) = e^{-(n-n_0)^2/\Delta^2} e^{i\varepsilon n} \mathbf{e_s}$ at site $m = 0$ in the synthetic frequency lattice, where $\mathbf{e_s} = (1,0)^T$ or $(0,1)^T$ represents the left or right side of the ladder where the excitation source is applied [see Fig. S1a]. ε is the quasienergy of the edge mode we want to excite. We apply the unitary step operator U to the initial state $|\mathbf{s}(n=0)\rangle$ to simulate light circulating in the ring for one roundtrip. We choose corresponding $\mathbf{e_s}$ and $\Delta = 0$, $n_0 = 10$, $\varepsilon = -0.27\ \Omega$ to excite the edge modes with quasienergy of $-0.27\ \Omega$ and plot the wave function distribution $P(m,n)$ after n-th step in Fig. S1b-S1e. One sees that if there is an excitation source at state $|m,p\rangle = |0,H\rangle$ [see Fig. S1b], the edge mode on the $|H\rangle$ side of the ladder with $\varepsilon = -0.27$ Ω is excited and evolves in the one-way manner, while almost no modes are excited on the

$|V\rangle$ side of the ladder [Fig. S1c], and vice versa. This indicates that the excited modes with single dispersion are all localized on one side of the ladder and propagate unidirectionally along the frequency dimension, exhibiting the characteristics of topological edge modes.

V. THE INTRODUCING OF BULK

We can use multiple ring resonators to introduce bulk in our model. For example, we can arrange multiple identical ring resonators in an array along the spatial dimension to form a second dimension in the spatial space. Bulk is then introduced as now the synthetic lattice holds more than 2 columns in a frequency-spatial space [17, 18].

VI. THE EXPECTED SCALING BEHAVIOR OF THE DIFFUSION DISTANCE

We simulate the evolution of the 1D random walk, 1D conventional DTQW, and 1D DTQW with different Γ in the frequency dimension, fitting the results to obtain their expected scaling behavior of the diffusion distance, as shown in Fig. S2a-S2c. The expected scaling behavior of random walks is $M(n) = \sqrt{n}$, while $M(n) = 0.54\ n$ for the conventional DTQW, which verifies that the conventional DTQW spreads quadratically faster than the classical random walk. For DTQW in the frequency dimension, the expected scaling behavior $M(n)$ is also linear. The slope of $M(n)$ is smaller than conventional DTQW in the weak modulation case, but when modulation strength increase, the slope quickly surpasses the conventional DTQW. In Fig. S2d, we show the linear relationship between the slope $M \cdot n^{-1}$ of DTQW in the frequency dimension and the modulation strength Γ: $M \cdot n^{-1} = 1.38\ (\pi\Gamma)^{-1}$, indicating that our model in the strong modulation case has a significant speed advantage.

VII. THE SIMULATION OF DTQWS WITH PERTURBATIONS

For the 1D conventional DTQW simulated in Fig. 3b in our manuscript, we add perturbations to polarization rotation θ, where a coefficient $(1+R\cdot\delta_1)$ is multiplied on θ. Here R is the random number generated in-between $(-0.5, 0.5)$, and δ_1 is a constant for labelling the amplitude of disorders. The results of the wave function distribution $P(m, n)$ with $\delta_1 = 0$,

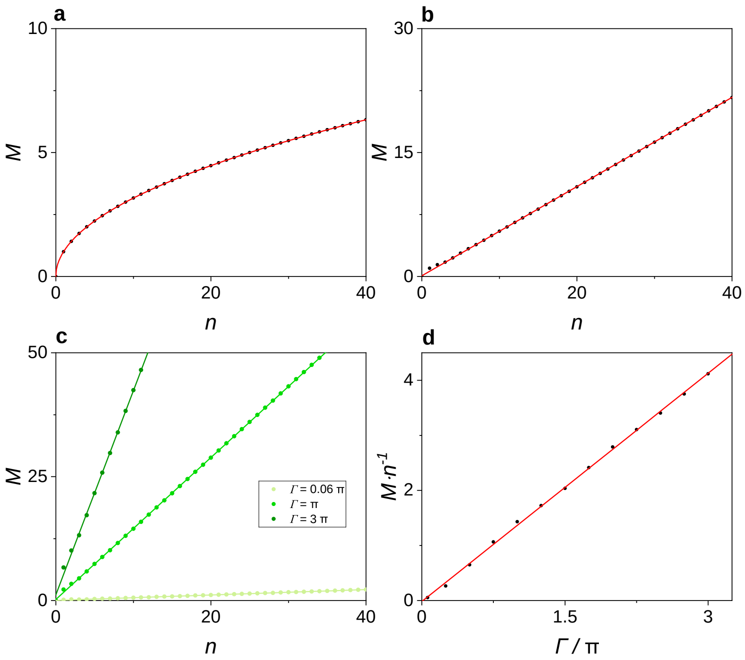

FIG. S2. **The expected scaling behavior.** The fitting of the variation of diffusion distances with the step numbers n: **a,** 1D random walk, $M(n) = \sqrt{n}$, **b,** 1D conventional DTQW, $M(n) = 0.54\ n$, **c,** 1D DTQW in the synthetic frequency dimension with different $\mathit{\Gamma}$, $M(n) = 0.05\ n$ for $\mathit{\Gamma} = 0.06\ \pi$, $M(n) = 1.43\ n$ for $\mathit{\Gamma} = \pi$, and $M(n) = 4.12\ n$ for $\mathit{\Gamma} = 3\ \pi$. **d,** The slope of the linear relationship between M and n of 1D DTQW in frequency space varied with the modulate strength $\mathit{\Gamma}$: $M \cdot n^{-1} = 1.38\ (\pi \mathit{\Gamma})^{-1}$

0.1, and 0.2 are shown in Fig. S3a-S3c. One can see that the peaks of $P(m, n)$ on both sides still exist with each case of δ_1, since the 1D conventional DTQW is protected by the non-zero winding number [19].

For the 1D DTQW in the frequency dimension with different modulation strength in the Fig. 3c-3e in our manuscript, we add perturbations to the polarization rotation θ, modulation phase ϕ_V, and the modulation strength $\mathit{\Gamma}$, where three coefficients of $(1+R\cdot\delta_1)$, $(1+R\cdot\delta_2)$, and $(1+R\cdot\delta_3)$ are add on each θ, ϕ_V, and $\mathit{\Gamma}$, respectively. The results are shown in Fig. S4a-S4i, Fig. S5a-S5i, and Fig. S6a-S6i. In the case of $\mathit{\Gamma} = 0.06\ \pi$, the influence of the perturbations on the wave function distribution $P(m, n)$ is not significant [see Fig. S4a-S4i]. The similar situation can also be seen in the strong modulation case [see Fig. S5a-S5i, and Fig. S6a-S6i]. Although there is a slight change of $P(m, n)$ in the middle part of the lattice [where the value of $P(m, n)$ is small] when δ_2 and δ_3 increase to

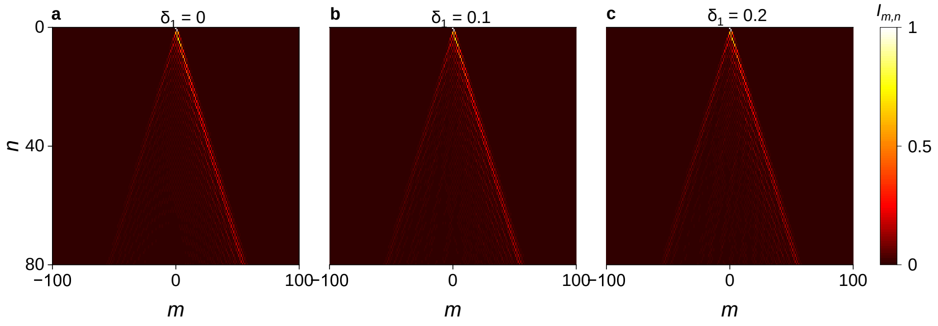

FIG. S3. **Evolution of 1D conventional DTQW.** Evolution of 1D conventional DTQW with perturbations on polarization rotation with **a,** $\delta_1 = 0$, **b,** $\delta_1 = 0.1$, **c,** $\delta_1 = 0.2$ in simulations.

0.2 [see Fig. S5f, Fig. S5i, Fig. S6f, and Fig. S6i], the overall distribution of $P(m, n)$ and the diffusion distances does not show significant changes.

Compared with the 1D conventional DTQWs, the 1D DTQW in the frequency dimension exhibits similar robustness against small disorder, showing that our model indeed retains the topological characteristic although the bulk is removed and under the strong modulation. These topological features can protect the quantum gate operations, enhancing their anti-interference capability.

VIII. THE EFFICIENCY OF THE QUANTUM WALK TRANSPORT IN THE SYNTHETIC FREQUENCY DIMENSION

The dynamics of the quantum walker is described by its wave function, so the efficiency of the transport is a statistical problem. The EOMs used here induce the jumping operatios between sidebands (resonant modes) following the Jacobi-Anger expansion with hopping (shifting) amplitudes dependent on Bessel functions (See Eq. (8) in the manuscript). The overall shifting over all modes is conserved if loss is ignored. To properly reflect the efficiency of the quantum walk transport in our proposal, one may use the return probability $P_{m=0}(n) = \Sigma_p |\langle 0, p|U^n|\varphi_0\rangle|^2$, which is defined as the probability for the walker to be still or again at the initial site (probability at the initial position), as a good measure to quantify the efficiency of the transport [20, 21]. The faster that $P_{m=0}(n)$ gets away from 1, the quantum transport exhibits high efficiency.

We plot the calculated return probability of a walker being found at the initial position

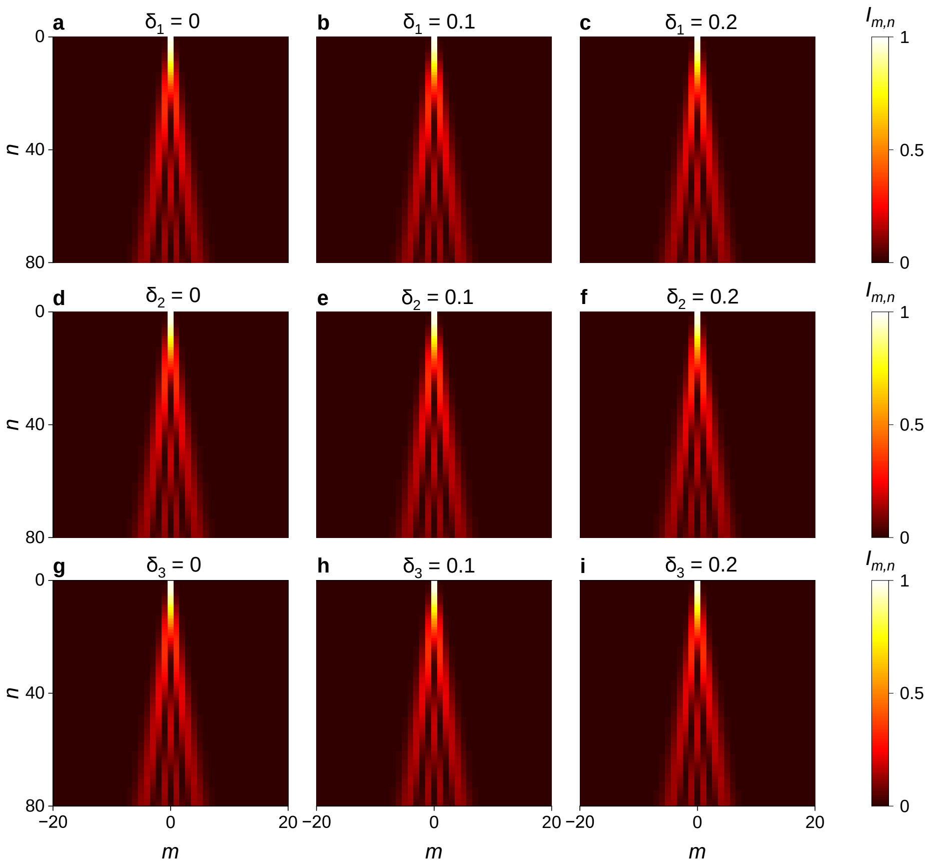

FIG. S4. **Evolution of 1D DTQW in the synthetic frequency dimension with perturbations.** Evolution of 1D DTQW in the synthetic frequency dimension with perturbations on **a,** θ with $\delta_1 = 0$, **b,** θ with $\delta_1 = 0.1$, **c,** θ with $\delta_1 = 0.2$, **d,** ϕ_V with $\delta_2 = 0$, **e,** ϕ_V with $\delta_2 = 0.1$, **f,** ϕ_V with $\delta_2 = 0.2$, **g,** $\mathit{\Gamma}$ with $\delta_3 = 0$, **h,** $\mathit{\Gamma}$ with $\delta_3 = 0.1$, **i,** $\mathit{\Gamma}$ with $\delta_3 = 0.2$, in simulations. The modulation strength $\mathit{\Gamma} = 0.06\ \pi$.

after n roundtrip in Fig. S7. All DTQWs and random walk have decreasing $P_{m=0(n)}$ as n increases, but follow different asymptotic lines. A 1D conventional quantum walker evolves away from the original site faster and is less likely to move back compared to the 1D DTQW in the frequency dimension with $\mathit{\Gamma} = \pi$ and $\mathit{\Gamma} = 3\ \pi$ evolve away from the initial position faster than conventional DTQW. Therefore, the effiviency of the transport with coherent multiple long-range couplings in our proposal under strong modulation is higher than a 1D conventional quantum walk.

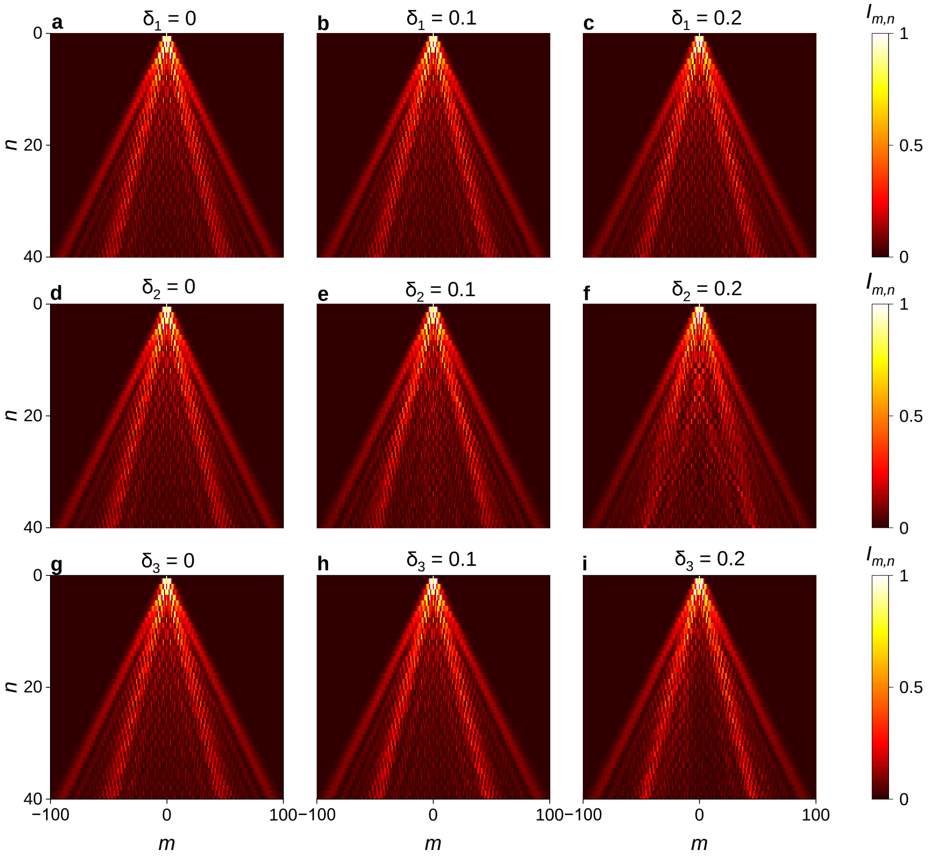

FIG. S5. **Evolution of 1D DTQW in the synthetic frequency dimension with perturbations.** Evolution of 1D DTQW in the synthetic frequency dimension with perturbations on **a,** θ with $\delta_1 = 0$, **b,** θ with $\delta_1 = 0.1$, **c,** θ with $\delta_1 = 0.2$, **d,** ϕ_V with $\delta_2 = 0$, **e,** ϕ_V with $\delta_2 = 0.1$, **f,** ϕ_V with $\delta_2 = 0.2$, **g,** Γ with $\delta_3 = 0$, **h,** Γ with $\delta_3 = 0.1$, **i,** Γ with $\delta_3 = 0.2$, in simulations. The modulation strength $\Gamma = \pi$.

IX. THE QUANTUM WALK ACCELERATION ON QUANTUM GATE OPERATION

The topological band in Eq. (8) in the main text and increase in DTQW diffusion speed shown in our model could help us significantly increase the operation speed of quantum gates. In Table S1, we compare the number of steps in DTQW required to achieve the target quantum-gate (Phase-shift gate and Hadamard gate) with the conventional DTQW method [22], 1D DTQW in the synthetic frequency dimension under weak modulation ($\Gamma = 0.01\,\pi$), and 1D DTQW under strong modulation ($\Gamma = 0.25\,\pi$ and $\Gamma = \pi$ for two gates, respectively).

In [22], the authors used DTQW to implement single-qubit gates, namely the phase-shift

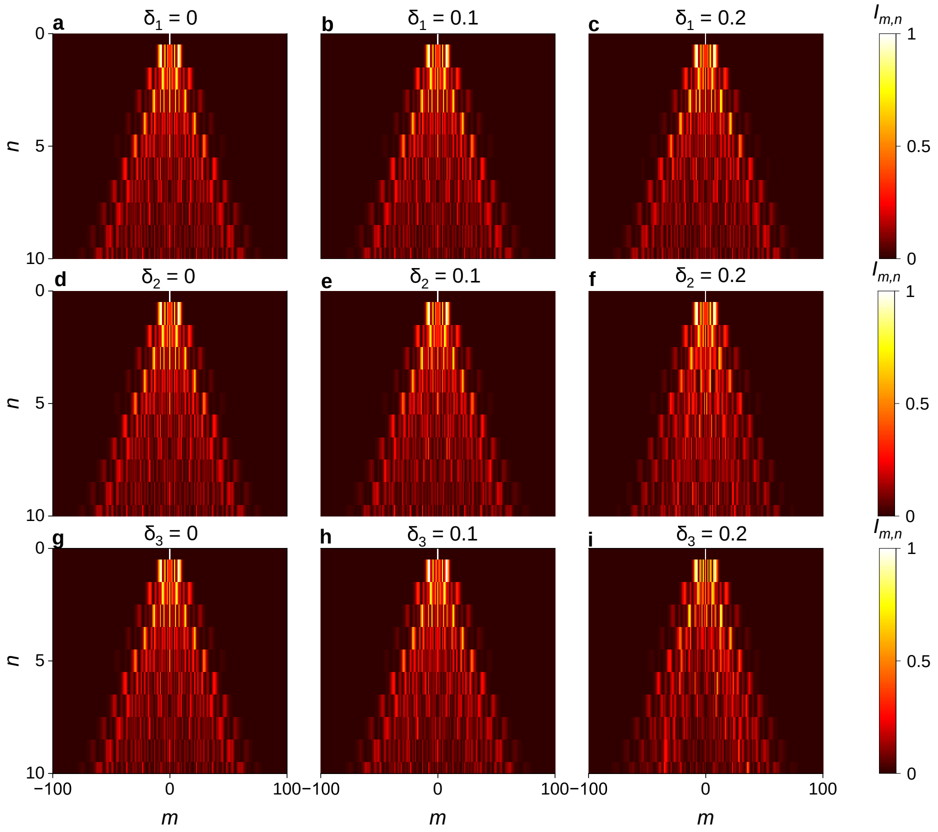

FIG. S6. **Evolution of 1D DTQW in the synthetic frequency dimension with perturbations.** Evolution of 1D DTQW in the synthetic frequency dimension with perturbations on **a,** θ with $\delta_1 = 0$, **b,** θ with $\delta_1 = 0.1$, **c,** θ with $\delta_1 = 0.2$, **d,** ϕ_V with $\delta_2 = 0$, **e,** ϕ_V with $\delta_2 = 0.1$, **f,** ϕ_V with $\delta_2 = 0.2$, **g,** Γ with $\delta_3 = 0$, **h,** Γ with $\delta_3 = 0.1$, **i,** Γ with $\delta_3 = 0.2$, in simulations. The modulation strength $\Gamma = 3\ \pi$.

gate and Hadamard gate, in 4 and 17 steps, respectively. In our proposal of the 1D DTQW in the synthetic frequency dimension under the weak modulation, e.g., $\Gamma = 0.01\ \pi$, these two quantum gate operations may be achieved by using the corresponding unitary step operator in the quasi-momentum space $U_k \equiv \int_{\mathrm{BZ}} dk M_U \otimes |k\rangle\langle k|$. The specific forms of M_U can then be written as:

$$M_U = \begin{pmatrix} e^{i\Gamma\cos(k+\phi_H)}\cos\frac{\theta}{2} & -e^{i\Gamma\cos(k+\phi_H)}\sin\frac{\theta}{2} \\ e^{i\Gamma\cos(k+\phi_V)}\sin\frac{\theta}{2} & e^{i\Gamma\cos(k+\phi_V)}\cos\frac{\theta}{2} \end{pmatrix}^{25}, \tag{S4}$$

for the phase-shift gate with $\Gamma = 0.01\ \pi$, $\cos(k+\phi_H) = 0$, $\cos(k+\phi_V) = -1$, and $\theta = 0$,

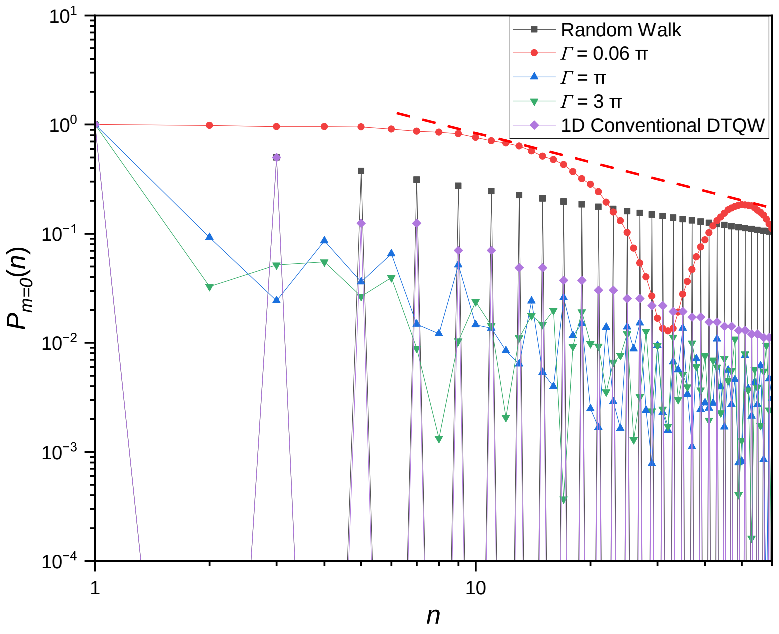

FIG. S7. **The return probability.** Return probability calculated in the classical random wlak (black), 1D conventional DTQW (purple), and 1D DTQW in the frequency dimension with three different Γ (red, blue, and green, respectively) versus step (roundtrip) number n. The dotted lines are the asymptotes of each curve.

and

$$M_U = \left[\begin{pmatrix} e^{i\Gamma\cos(k+\phi_{H_2})}\cos\frac{\theta_2}{2} & -e^{i\Gamma\cos(k+\phi_{H_2})}\sin\frac{\theta_2}{2} \\ e^{i\Gamma\cos(k+\phi_{V_2})}\sin\frac{\theta_2}{2} & e^{i\Gamma\cos(k+\phi_{V_2})}\cos\frac{\theta_2}{2} \end{pmatrix}^{100} \begin{pmatrix} e^{i\Gamma\cos(k+\phi_{H_1})}\cos\frac{\theta_1}{2} & -e^{i\Gamma\cos(k+\phi_{H_1})}\sin\frac{\theta_1}{2} \\ e^{i\Gamma\cos(k+\phi_{V_1})}\sin\frac{\theta_1}{2} & e^{i\Gamma\cos(k+\phi_{V_1})}\cos\frac{\theta_1}{2} \end{pmatrix}^{100} \right]^{201}, \tag{S5}$$

for the Hadamard gate with $\Gamma = 0.01\ \pi$, $\cos(k+\phi_{H_1}) = 1$, $\cos(k+\phi_{V_1}) = 1$, $\theta_1 = -\pi/2$, $\cos(k+\phi_{H_2}) = 0$, $\cos(k+\phi_{V_2}) = 1$, and $\theta_2 = 0$. It shows that this model requiresn 25 and 20301 steps (roundtrips) to implement the phase-shif gate and Hadamard gate, respectively.

However, these two quantum gate operations can be implemented with 1 step (roundtrip) using the 1D DTQW under the strong modulation in our model, as shown in our manuscript. In particular, we set $\theta = 0$, $\Gamma\cos(k+\phi_H) = 0$, and $\Gamma\cos(k+\phi_V) = \pi/4$ in Eq. (6) in the main text, so the corresponding operator $U_k \equiv \int_{\mathrm{BZ}} M_U \otimes |k\rangle\langle k|$ has $M_U = \begin{pmatrix} 1 & 0 \\ 0 & e^{i\frac{\pi}{4}} \end{pmatrix}$, which is the phase-shift gate for qubits with definite quasi-momentum $|\phi_1, \phi_2\rangle \otimes |k\rangle$. For Hadamard gate, we set $\theta = -\pi/2$, $\Gamma\cos(k+\phi_H) = 0$, and $\Gamma\cos(k+\phi_V) = \pi$, so the corresponding

TABLE S1. The number of steps in DTQW required to achieve Phase-shift gate and Hadamard gate with conventional DTQW method, and 1D DTQW in the synthetic dimension under either weak modulation or strong modulation.

	1D conventional DTQW	Weak modulation case ($\mathit{\Gamma} = 0.01\pi$)	Strong modulation case ($\mathit{\Gamma} = \pi/4$ or π)
Phase-shift (R_z) gate	4	25	1
Hadamard (H) gate	17	20301	1

$M_U = \begin{pmatrix} 1 & 1 \\ 1 & -1 \end{pmatrix}$. Therefore, Eq. (6) in the main text and its correspongding band with coherent multiple long-range translations greatly reduces the step in DTQW required for preparing quantum gates, which may not be fulfilled for the same model under the weak modulation.

Moreover, the fidelity of the X gate is calculated by $F_g = 1 - ||M_t - M_o||^2_{\text{HS}} = 1 - \text{Tr}[(M_t^\dagger - M_o^\dagger)(M_t - M_o)]$ in the main text and results in $1 - F_g \sim 10^{-30}$. The difference between F_g and 1 is small because we only retain the polarization components etangled with the given k when converting the resulting field distribution to the quasi-momentum space. The error in polarizations is mainly induced by, and entangled with, the part of the initial excitation whose quasi-momentum is not equal to the given k, which is ignored by the converting process.

X. THE REDUCING OF THE FIDELITIES OF THE ARBITRARY SINGLE QUBIT STATES

In fact, we use the qubit wavefunction in coordinate space (i.e., the frequency space here) to calculate the fidelity. We first transform the initial qubit $|H\rangle \otimes |k\rangle$ into frequency space to obtain its wavefunction, and then modulate this wavefunction in frequency space to reach the wavefunction $|\psi_o\rangle$ of output qubit in coordinate space. In calculation, we transform the target qubit and output qubit with the same k in the quasi-momentum space to the frequency space, obtaining the wavefunction $|\psi_t\rangle$ in the synthetic frequency dimension, and then using the equation $F = |\langle\psi_o|\psi_t\rangle|^2$ in our manuscript to calculate the fidelity. Since

the number of the frequency modes we used in simulation is finite, the distribution of the wavefunction in the frequency dimension actually extends beyond the frequency range under our considerations, which mainly causes the fidelity $F < 1$.

According to Table I in our manuscript, we choose $\theta = 0$, $\Gamma \cos(k + \phi_H) = 0$, and $\Gamma \cos(k + \phi_V) = \varphi$ to implement the phase-shift (R_z) gate operation, where φ is the phase-shift. In simulation, we set $\Gamma = \varphi$, $\cos(k + \phi_H) = 0$, and $\cos(k + \phi_V) = 1$, which indicates that the modulation strength of the DTQW is linearly dependent on φ. Therefore, as φ increases, the modulation strength of our model also increases, meaning that the long-range couplings are enriched, and the probability of walkers diffuse to the section outside the frequency range under our considerations also increases. It causes the fidelity reducing with the choice of φ increasing. One can see the fidelities in Fig. 4a-4c decrease when the phase-shift of the second phase-shift gate increase (the phase-shift of the first phase-shift gate is fixed to be unchanged), which is attributed to the finite frequency range and the strong modulation in simulations.

XI. THE REALISTIC ESTIMATE FOR THE FIDELITY OF THE QUBITS

In our model, the influence on fidelity mainly comes from the loss η_1 in the coupling between the resonant ring and the waveguide, η_2 in the polarization rotator, η_3 in the polarization splitter, and η_4 in the EOMs. These losses mainly influence the intensity to reduce the fidelity. According to Ref. [23], loss in the coupling between resonant rings and waveguides is roughly $\eta_1 \sim 0.1$ %. For the polarization rotator in Ref. [24], $\eta_2 \sim 1.5$ % can be estimated from the polarization conversion efficiencies. The work in Ref. [25] uses only polarization beam splitter to implement quantum gates and obtain $\eta_3 \sim 0.3$ %. The loss of an on-chip EOM [26] is ~ 0.1 %. Therefore, the fidelity for potential experiments should be corrected by $F = \left| \langle \psi_o | \sqrt{\prod_{i=1}^{4} (1 - \eta_i)} | \psi_t \rangle \right|^2 \sim 97$ %.

XII. THE TRADE-OFF BETWEEN THE NUMBER OF RESONATORS AND FIDELITY

Since the reducing of the fidelity are mainly indicated by the loss in the devices, the fidelity of N resonators can be calculated by $F = \left| \langle \psi_o | \left[\sqrt{\prod_{i=1}^{4} (1 - \eta_i)} \right]^N | \psi_t \rangle \right|^2$. The work

in Ref. [27] implement an entangled two-qubit Bell state with a state fidelity of 0.88, because of which we set 85% as the lowest acceptable level of fidelity in practical applications. When the number of the resonators increases to $N = 5$, the fidelity is $F \sim 0.859$, and when $N = 6$, $F \sim 0.833$. Hence in practical applications, a maximum of 5 resonant rings can be introduced.

XIII. THE REASON FOR USING ONLY ONE EOM TO INDUCE LONG-RANGE HOPPING

Multiple EOMs or a single modulation signal composed of multiple sinusoid functions at different frequencies may serve the long-range purpose. Previous examples usually focus on the weak modulation limit. Here we choose to use only one EOM but in the strong modulation limit, where we find it support not only coherent multiple long-range connectivities but also can provide simplified experiment proposal.

To explicitly see this fact, we give further explanations. Under the weak modulation limit, a single sinusoid function in the signal applied in EOM can only provide one type of long-range coupling with the length of $n\times$FSR (n times of FSR), therefore the number of the induced long-range hopping types is finite. For example, the work in Ref. [28] using three EOMs to add three types long-range couplings with the length of $1\times$FSR, $2\times$FSR, and $3\times$FSR, respectively. But in our model, an EOM in the strong modulation case can theoretically induce all types of the long-range hopping into the frequency lattice simultaneously, implement the coherent multiple long-range hopping. In the Eq. (6) in our manuscript, one can see Bessel functions of all orders, implying the emergence of the coherent multiple long-range hopping, which is the specific feature of the dynamic in the strong modulation case. Moreover, using only one EOM reduces the number of experimental parameters, being beneficial for simplifying the experiment.

XIV. REQUIREMENTS FOR INITIAL STATE PREPARATIONS AND MEASUREMENTS

The initial quantum states can be prepared by an on-chip heralded single photon source [29] together with a polarizaiton state generator [30]. An external waveguide is coupled with

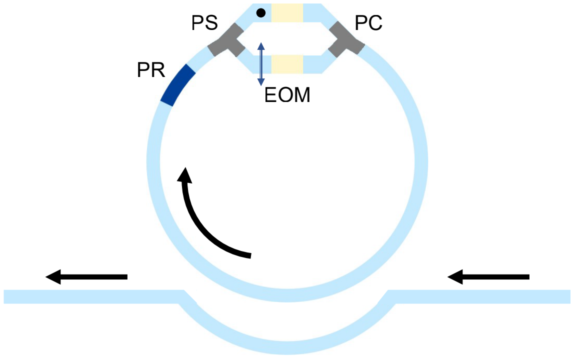

FIG. S8. **The structure of the ring resonator.** A ring resonator with a polarization rotator (PR), a polarizaition splitter (PS), two EOMs, and a polarization combiner (PC) coupled to a waveguide through a beam splitter (BS). The dots and black arrows represent H- and V-polarization respectively.

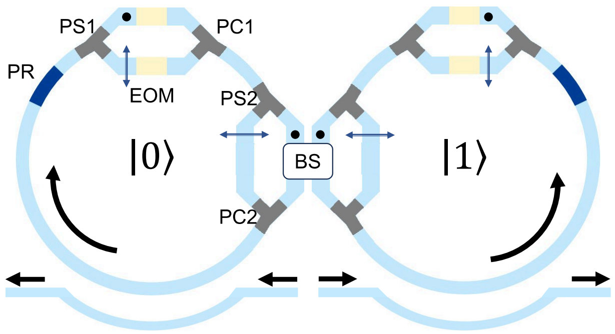

FIG. S9. **A CNOT gate consisting of two ring resonators.** A CNOT gate consisting of two ring resonators with polarization rotator (PR), polarization splitter (PS), EOMs, polarization combiner (PC), and a 1 : 1 beam splitter (BS). The dots and black arrows represent H- and V-polarization respectively.

the ring, where the photons are input into the ring from the through-port and the detection can be made at the drop-port waveguide (see Fig. S8) [23, 31]. In particular, following realistic experimental implementation in Ref. [23], one desires that the coupling waveguide has an optimized wrap-around geometry placed aside with the resonator to couple light out of each mode of the resonator with the same efficiency for the entire bandwidth of the synthetic dimension. The modulated photons are output through the coupled waveguide and selected by a tunable bandpass filter. The outputs of the filter are then sent to a superconducting nanowire single-photon detectors for measurements [23].

As an example, we prepose the potential experimental setup for CNOT gates, which is shown in Fig. S9. A single photon with specific polarization state is prepared by an on-chip heralded single photon source [29] together with a polarization state generator [30], and then sent to one of the two resonators to generate the initial two-qubit state. Two external waveguides are coupled with one of the rings respectively, where the photons are input into the ring from one of the through-port and the detection can be made at the both two drop-port waveguides [23, 31]. Following realistic experimental implementation in Ref. [23], one desires that the two coupling waveguides both has the same optimized wrap-around geometry placed aside with the respective resonator to couple light out of each mode of the resonator with the same efficiency for the entire bandwidth of the synthetic dimension. The modulated photons are output through the coupled waveguides and selected by two tunable bandpass filters. The outputs of the filters are then sent to two superconducting nanowire single-photon detectors for measurements [23].

XV. THE MATERIAL AND STRUCTURE OF THE RING RESONATOR

The ring resonator can be fabricated on the thin-film lithium niobite chip as a microresonator [23]. The resonator width of 1.5 μm and 50 % etching depth of 300 nm are adjusted to set the zero-dispersion wavelength of the fundamental TE mode at the center of the generated spectrum around 1550 nm. The resonator modes in the telecom wavelength region can be critically coupled with an intrinsic quality-factor of 10^6, and modes with wavelengths on the near-infrared region have an intrinsic quality of 3×10^5.

XVI. THE 2D DTQW IN THE FREQUENCY SPACE

Our proposal for implementing the 2D DTQW is shown in Fig. S10. Based on the ring resonator of the 1D DTQW in the frequency dimension, we add another pair of branches with a polarization rotator, a polarization splitter, two EOMs, and a polarization combiner. The modulation of the EOMs on the new branches is

$$D'(t) \equiv \begin{pmatrix} e^{i\Gamma \cos(K\Omega+\phi_H)} & 0 \\ 0 & e^{i\Gamma \cos(K\Omega+\phi_V)} \end{pmatrix} , \tag{S6}$$

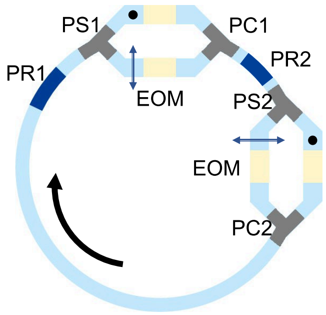

FIG. S10. **Ring resonator for 2D DTQW in the frequency space.** Ring resonator with two polarization rotators (PR1, PR2), two polarization splitters (PS1, PS2), four EOMs and two polarization combiners (PC1, PC2). The dots and black arrows represent H- and V-polarization respectively.

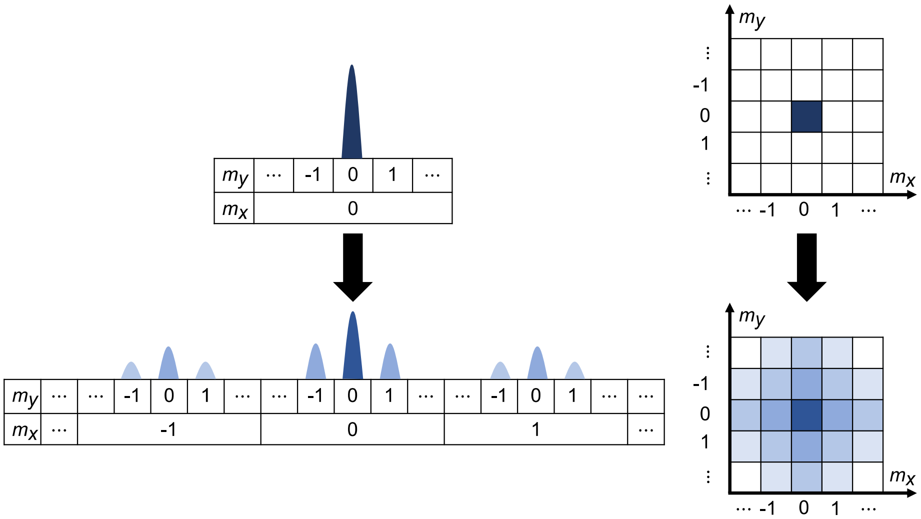

FIG. S11. **The 2D DTQW in the synthetic frequency space.** The initial position of the walker is $(0, 0)$. After one step of the 2D DTQW, the wave function distribution of the walker diffuses in the synthetic frequency space. The ellipsis in the table represent the lattice sites connected with the position $(0, 0)$ by coherent multiple long-range couplings.

where K is an integer satisfying $K >> 1$ and other parameters are set to be the same as the first branches or different.

The new EOMs generate a new synthetic frequency dimension where the spacing between lattice sites is $K\Omega$. We set the frequency dimension with spacing $K\Omega$ as the m_x axis, and another as the m_y axis. The one step of the DTQW of a walker with initial position $(0, 0)$

in these two axes is shown in Fig. S11, which is a 2D DTQW in the synthetic frequency space n_x-n_y extended from our 1D model.

The unitary step operator of the 2D DTQW in the frequency space is

$$U = T_y R(\theta_2) T_x R(\theta_1), \tag{S7}$$

$$R(\theta_1) = e^{-i\theta_1 \sigma_y/2}, \tag{S8}$$

$$R(\theta_2) = e^{-i\theta_2 \sigma_y/2}, \tag{S9}$$

$$\begin{aligned} T_x = &\sum_{m_x,l=\infty}^{\infty} i^l J_l(\Gamma) e^{il\phi_H} |m_x + l\rangle\langle m_x| \otimes |H\rangle\langle H| \\ &+ \sum_{m_x,l=\infty}^{\infty} i^l J_l(\Gamma) e^{il\phi_V} |m_x + l\rangle\langle m_x| \otimes |V\rangle\langle V|, \end{aligned} \tag{S10}$$

$$\begin{aligned} T_y = &\sum_{m_y,l=\infty}^{\infty} i^l J_l(\Gamma) e^{il\phi_H} |m_y + l\rangle\langle m_y| \otimes |H\rangle\langle H| \\ &+ \sum_{m_y,l=\infty}^{\infty} i^l J_l(\Gamma) e^{il\phi_V} |m_y + l\rangle\langle m_y| \otimes |V\rangle\langle V|, \end{aligned} \tag{S11}$$

XVII. THE TWO PHOTONS CASE WITH PHOTON-PHOTON INTERACTION

As an example, according to work in Ref. [31], we show a local photon-photon interaction in our model in the two photons case. The photon-photon interaction can be implemented by $\chi^{(3)}$ processes in the waveguide forming the ring, which is shown in Fig. S12. The main part of the ring resonator consists two sections of waveguides, which has the same $\chi^{(3)}$ nonlinear susceptibility but opposite group velocity dispersion (GVD) (see Fig. S12). For linear light propagation, this ring acts as the same as the ring with zero GVD. For nonlinear propagation, there are only self-phase modulation (SPM) and cross-phase modulation (XPM) process in the waveguide, and it can be reduced to only including the SPM process in any Hilbert space with a fixed photon number [31].

According to the Eq. (6)-(7) in our manuscript, the propagation in the ring without nonlinear process is

$$a_m(t + T_R) = J_0(\Gamma) R(\theta) a_m(t) + \sum_{l=-\infty}^{\infty} i^l J_l(\Gamma) [d_l R(\theta) a_{m-l}(t) + d_{-l} R(\theta) a_{m+l}(t)], \tag{S12}$$

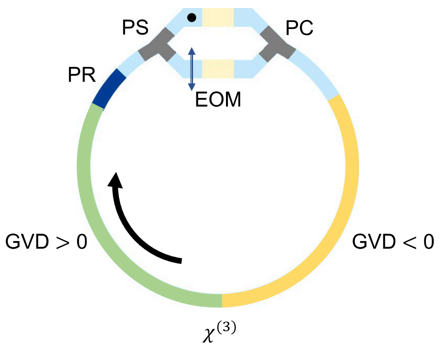

FIG. S12. **Ring resonator for two photons case.** Ring resonator with a polarization rotator (PR), a polarization splitter (PS), two EOMs, a polarization combiner (PC). The main part of the ring is composed by two types of single-mode waveguides (green and yellow). The dots and black arrows represent H- and V-polarization respectively.

$$d_l = \begin{pmatrix} e^{il\phi_H} & 0 \\ 0 & e^{il\phi_V} \end{pmatrix} . \tag{S13}$$

When considering the nonlinear process, Eq. (S12) becomes

$$\begin{aligned} a_m(t+T_R) =& J_0(\mathit{\Gamma})R(\theta)a_m(t) + \sum_{l=-\infty}^{\infty} i^l J_l(\mathit{\Gamma})[d_l R(\theta)a_{m-l}(t) + d_{-l}R(\theta)a_{m+l}(t)], \\ &+ i\frac{\omega_0^2\Omega}{4\pi\beta_0 c^2}\chi^{(3)}|a_m(t)|^2 a_m(t) + i\sum_{n\neq m}\frac{\omega_0^2\Omega}{\pi\beta_0 c^2}\chi^{(3)}|a_n(t)|^2 a_m(t), \end{aligned} \tag{S14}$$

where β_0 denotes the propagation wavevector of frequency mode ω_0 [31]. The penultimate term represents the SPM process, which can be regarded as the on-site interaction term, and the last term is the XPM process. When fix the photon number in our model, the XPM term converts to the SPM term, and the unitary step operator can be written as

$$U = TR(\theta) - U_i, \tag{S15}$$

where U_i represents the on-site interaction and $U_i = i\frac{\omega_0^2\Omega}{4\pi\beta_0 c^2}\chi^{(3)}|a_m(t)|^2$.

[1] M. A. Broome, A. Fedrizzi, B. P. Lanyon, I. Kassal, A. Aspuru-Guzik, and A. G. White, Discrete single-photon quantum walks with tunable decoherence, Phys. Rev. Lett. **104**, 153602 (2010).

[2] F. Cardano, A. D'Errico, A. Dauphin, M. Maffei, B. Piccirillo, C. de Lisio, G. De Filippis, V. Cataudella, E. Santamato, L. Marrucci, M. Lewenstein, and P. Massignan, Detection of zak phases and topological invariants in a chiral quantum walk of twisted photons, Nat. Commun. **8**, 15516 (2017).

[3] A. D'Errico, F. Cardano, M. Maffei, A. Dauphin, R. Barboza, C. Esposito, B. Piccirillo, M. Lewenstein, P. Massignan, and L. Marrucci, Two-dimensional topological quantum walks in the momentum space of structured light, Optica **7**, 108 (2020).

[4] F. D. Colandrea, A. Babazadeh, A. Dauphin, P. Massignan, L. Marrucci, and F. Cardano, Ultra-long quantum walks via spin–orbit photonics, Optica **10**, 324 (2023).

[5] A. Schreiber, K. N. Cassemiro, V. Potoček, A. Gábris, P. J. Mosley, E. Andersson, I. Jex, and C. Silberhorn, Photons walking the line: A quantum walk with adjustable coin operations, Phys. Rev. Lett. **104**, 050502 (2010).

[6] A. Schreiber, A. Gábris, P. P. Rohde, K. Laiho, M. Štefaňák, V. Potoček, C. Hamilton, I. Jex, and C. Silberhorn, A 2d quantum walk simulation of two-particle dynamics, Science **336**, 55 (2012).

[7] D. Bagrets, K. W. Kim, S. Barkhofen, S. De, J. Sperling, C. Silberhorn, A. Altland, and T. Micklitz, Probing the topological anderson transition with quantum walks, Phys. Rev. Res. **3**, 023183 (2021).

[8] P. Held, M. Engelkemeier, S. De, S. Barkhofen, J. Sperling, and C. Silberhorn, Driven gaussian quantum walks, Phys. Rev. A **105**, 042210 (2022).

[9] J. Boutari, A. Feizpour, S. Barz, C. D. Franco, M. S. Kim, W. S. Kolthammer, and I. A. Walmsley, Large scale quantum walks by means of optical fiber cavities, J. Opt. **18**, 094007 (2016).

[10] L. Sansoni, F. Sciarrino, G. Vallone, P. Mataloni, A. Crespi, R. Ramponi, and R. Osellame, Two-particle bosonic-fermionic quantum walk via integrated photonics, Phys. Rev. Lett. **108**, 010502 (2012).

[11] M. Gräfe, R. Heilmann, M. Lebugle, D. Guzman-Silva, A. Perez-Leija, and A. Szameit, Integrated photonic quantum walks, J. Opt. **18**, 103002 (2016).

[17] L. Yuan, Y. Shi, and S. Fan, Photonic gauge potential in a system with a synthetic frequency dimension, Opt. lett. **41**, 741 (2016).

[13] L. Yuan and S. Fan, Bloch oscillation and unidirectional translation of frequency in a dynam-

ically modulated ring resonator, Optica **3**, 1014 (2016).

[14] L. Yuan, A. Dutt, and S. Fan, Synthetic frequency dimensions in dynamically modulated ring resonators, APL Photonics **6** (2021).

[15] A. Dutt, Q. Lin, L. Yuan, M. Minkov, M. Xiao, and S. Fan, A single photonic cavity with two independent physical synthetic dimensions, Science **367**, 59 (2020).

[16] D. Hügel and B. Paredes, Chiral ladders and the edges of quantum hall insulators, Physical Review A **89**, 023619 (2014).

[17] L. Yuan, Y. Shi, and S. Fan, Photonic gauge potential in a system with a synthetic frequency dimension, Optics letters **41**, 741 (2016).

[18] T. Ozawa, H. M. Price, N. Goldman, O. Zilberberg, and I. Carusotto, Synthetic dimensions in integrated photonics: From optical isolation to four-dimensional quantum hall physics, Physical Review A **93**, 043827 (2016).

[19] T. Kitagawa, M. S. Rudner, E. Berg, and E. Demler, Exploring topological phases with quantum walks, Physical Review A—Atomic, Molecular, and Optical Physics **82**, 033429 (2010).

[20] O. Mülken and A. Blumen, Efficiency of quantum and classical transport on graphs, Phys. Rev. E **73**, 066117 (2006).

[21] X.-P. Xu, Continuous-time quantum walks on one-dimensional regular networks, Phys. Rev. E **77**, 061127 (2008).

[22] N. B. Lovett, S. Cooper, M. Everitt, M. Trevers, and V. Kendon, Universal quantum computation using the discrete-time quantum walk, Phys. Rev. A **81**, 042330 (2010).

[23] U. A. Javid, R. Lopez-Rios, J. Ling, A. Graf, J. Staffa, and Q. Lin, Chip-scale simulations in a quantum-correlated synthetic space, Nat. Photonics **17**, 883 (2023).

[24] D. Shahwar, M. Kapulainen, T. Haatainen, M. Harjanne, M. Cherchi, Z. Sun, T. Aalto, and K. Tappura, On-chip polarization rotators using metasurface, ACS omega **9**, 33055 (2024).

[25] G.-L. Jiang, J.-B. Yuan, W.-Q. Liu, and H.-R. Wei, Efficient and deterministic high-dimensional controlled-swap gates on hybrid linear optical systems with high fidelity, Physical Review Applied **21**, 014001 (2024).

[26] H.-H. Lu, J. M. Lukens, N. A. Peters, O. D. Odele, D. E. Leaird, A. M. Weiner, and P. Lougovski, Electro-optic frequency beam splitters and tritters for high-fidelity photonic quantum information processing, Physical review letters **120**, 030502 (2018).

[27] A. Bhattacharjee, P. Jain, J. Deshmukh, S. Das, M. Chand, M. P. Patankar, and R. Vijay,

Demonstration of two qubit entangling gates in a 2d ring resonator based coupler architecture, Scientific Reports **15**, 4426 (2025).

[28] H. X. Dinh, A. Balčytis, T. Ozawa, Y. Ota, G. Ren, T. Baba, S. Iwamoto, A. Mitchell, and T. G. Nguyen, Reconfigurable synthetic dimension frequency lattices in an integrated lithium niobate ring cavity, Communications Physics **7**, 185 (2024).

[29] S. Signorini and L. Pavesi, On-chip heralded single photon sources, AVS Quantum Sci. **2** (2020).

[30] D. Cheng, K. Wang, C. Roques-Carmes, E. Lustig, O. Y. Long, H. Wang, and S. Fan, Non-abelian lattice gauge fields in photonic synthetic frequency dimensions, Nature **637**, 52 (2025).

[31] L. Yuan, A. Dutt, M. Qin, S. Fan, and X. Chen, Creating locally interacting hamiltonians in the synthetic frequency dimension for photons, Photon. Res. **8**, B8 (2020).
